# The surface chemistry of a nanocellulose drug carrier unravelled by MAS-DNP†

**DOI:** 10.1039/c9sc06312a

**Published:** 2020-03-13

**Authors:** Akshay Kumar, Hippolyte Durand, Elisa Zeno, Cyril Balsollier, Bastien Watbled, Cecile Sillard, Sébastien Fort, Isabelle Baussanne, Naceur Belgacem, Daniel Lee, Sabine Hediger, Martine Demeunynck, Julien Bras, Gaël De Paëpe

**Affiliations:** Univ. Grenoble Alpes, CEA, CNRS, IRIG-MEM Grenoble France gael.depaepe@cea.fr; Univ. Grenoble Alpes, CNRS, Grenoble-INP, LGP2 Grenoble France julien.bras@pagora.grenoble-inp.fr; Centre Technique du Papier (CTP) Grenoble France; Univ. Grenoble Alpes, CNRS, CERMAV Grenoble France; Univ. Grenoble Alpes, CNRS, DPM Grenoble France

## Abstract

Cellulose nanofibrils (CNF) are renewable bio-based materials with high specific area, which makes them ideal candidates for multiple emerging applications including for instance on-demand drug release. However, in-depth chemical and structural characterization of the CNF surface chemistry is still an open challenge, especially for low weight percentage of functionalization. This currently prevents the development of efficient, cost-effective and reproducible green synthetic routes and thus the widespread development of targeted and responsive drug-delivery CNF carriers. We show in this work how we use dynamic nuclear polarization (DNP) to overcome the sensitivity limitation of conventional solid-state NMR and gain insight into the surface chemistry of drug-functionalized TEMPO-oxidized cellulose nanofibrils. The DNP enhanced-NMR data can report unambiguously on the presence of trace amounts of TEMPO moieties and depolymerized cellulosic units in the starting material, as well as coupling agents on the CNFs surface (used in the heterogeneous reaction). This enables a precise estimation of the drug loading while differentiating adsorption from covalent bonding (∼1 wt% in our case) as opposed to other analytical techniques such as elemental analysis and conductometric titration that can neither detect the presence of coupling agents, nor differentiate unambiguously between adsorption and grafting. The approach, which does not rely on the use of ^13^C/^15^N enriched compounds, will be key to further develop efficient surface chemistry routes and has direct implication for the development of drug delivery applications both in terms of safety and dosage.

## Abstract 2

DNP-enhanced solid-state NMR unravels the surface chemistry of functionalized nanocellulose.

## Graphical abstract

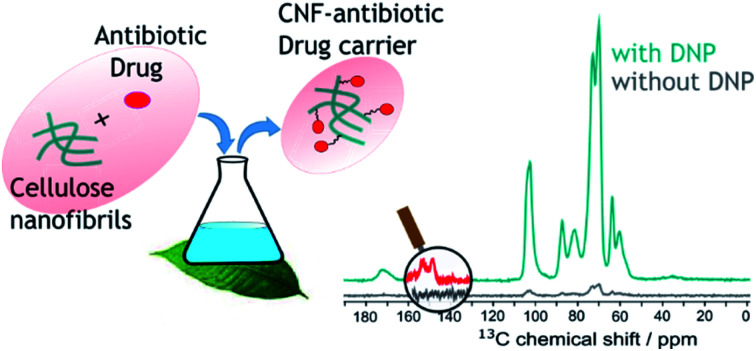

## Introduction

Wood-derived cellulose finds industrial applications in a wide range of fields, from paper products to buildings, cosmetics, foodstuffs or medical industry,^1,2^ with numerous kinds of industrially produced cellulosic materials. An important milestone was achieved by Turbak and co-workers in the 1980s with the introduction of a new type of cellulosic material, called cellulose nanofibrils (CNFs),^3–5^ which triggered a strong and lasting scientific enthusiasm. Indeed, in addition to the characteristics inherited from cellulose, such as widespread availability, biodegradability and biocompatibility, CNFs also present excellent mechanical properties, 5 to 20 nm width fibers and a high specific area (∼100 m^2^ g^−1^) that results in extended tunable surface chemistry.^6^ CNFs can be organized into different 2D and 3D nano-structures such as films, membranes, hydrogels or aerogels. At the end of the 2000s, a particular grade of CNF was designed by pretreating the cellulose fibers by means of a TEMPO-mediated oxidation.^7,8^ These TEMPO-oxidized CNFs (CNF-t) bear aldehyde and carboxylic acid groups at the nanofiber surface that offer new opportunities for functionalization strategies.

Drug delivery is one of the promising fields of application for CNF-t in the biomedical industry.^9^ For example, equivalent performances to commercial products were obtained in 2016 for CNF-t gel formulations containing five times less ibuprofen, providing a proof of concept for the efficiency of drug delivery for such systems.^10^ In 2017, the release profile for six active pharmaceutical ingredients (APIs) incorporated in CNF-t hydrogels was also investigated, demonstrating a stable behavior towards freeze-drying and subsequent successful rehydration.^11^ Notably, one of the drugs studied, an antibacterial compound metronidazole was further tested in interaction with different cellulose derivatives in order to better control its release.^12^ At this stage it is important to note that these studies only relied so far on drug adsorption mechanisms. It is thus important to investigate the loading of drugs, such as metronidazole, onto CNF-t through covalent binding with the aim to provide a better-controlled drug release on-demand.

Covalent binding of molecules onto cellulosic nanomaterials is currently an intensive field of research. Functional linkers can be covalently grafted to the CNF surface through *e.g.* etherification, amidation, esterification, and sialylation.^13–16^ These approaches mostly rely so far on the use of organic solvents such as DMF/DMSO and optimized reagents such as pyridine, carbodiimides, *etc.* to induce grafting at the C6 position on the CNF.^15^ Subsequent steps typically involve click-chemistry reactions, as described by Sharpless *et al.* in 2001, to further attach on to the linker an additional molecule of interest. Within this context, Diels–Alder reactions, which are compatible with these principles, have been recently implemented to produce new materials, using in particular the furan–maleimide strategy.^17,18^ In the biomedical context, the Diels–Alder reaction has also been used to immobilize multicolor fluorescent probes onto CNF for biological imaging^16^ and to produce enzymatically activated oligosaccharide-prodrugs of doxorubicin.^19^

Nevertheless, access to unambiguous information regarding the surface chemistry of this type of system is currently lacking. This has recently been discussed in detail by Foster *et al.*^20^ Standard techniques (FTIR, solid-state NMR, XPS) do not provide enough sensitivity and resolution to differentiate between low levels of adsorption and grafting or to understand reliably the surface chemistry of these key materials.^20^ Elemental analysis (N, C, and O) and conductometric titration are often the only techniques available to indirectly probe surface modifications. In addition, and in line with a sustainable green strategy compatible with ecological concerns and medical applications, it is becoming increasingly important to perform these surface reactions in aqueous conditions employing readily available reagents/catalysts with minimal sensitivity to water. This further decreases the kinetic and activation efficiency of the carboxylic groups compared to the use of organic solvent.^21^ All in all, the lack of techniques able to report on low level of CNF surface modifications impedes the development of efficient, cost-effective and reproducible green synthetic routes and thus the widespread development of targeted and responsive drug-delivery CNF carriers.

In this work, we show that dynamic nuclear polarization (DNP) enhanced solid-state NMR (ssNMR) can be used to unravel the surface chemistry of an innovative nanocellulose drug carrier even in the case of a very low level of grafting (<1 wt%), currently well beyond reach for all other techniques.

**Fig. 1:**
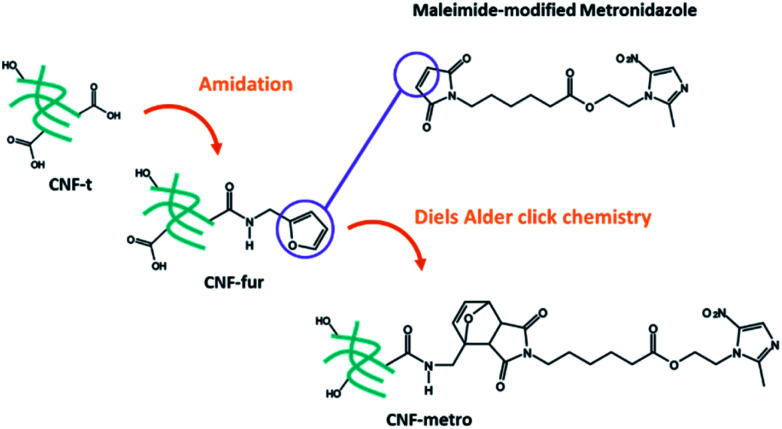
General multistep immobilization procedure of maleimide-modified metronidazole on CNF-t.

More precisely we report on the implementation of a Diels–Alder reaction under heterogeneous aqueous conditions aiming at grafting the metronidazole drug onto CNF. CNFs-t were first amidated with furfurylamine in order to functionalize them with pending furan groups. Metronidazole was chemically modified with a maleimide derived carboxylic acid in order to introduce an ester function between the drug and the maleimide ring. The Diels–Alder reaction between the furan functionalized CNF-t (CNF-fur) and metronidazole containing maleimide, as depicted in Fig. 1, was triggered by heat. The ester function present on the linking chain has been chosen for its known sensitivity to enzymatic or chemical hydrolysis.^22–24^ Indeed, esterases are ubiquitous enzymes, available in fat tissues and at infection sites.^25^ This new CNF-t based complex represents thus a smart drug carrier formulation with “on-demand” API release abilities in the presence of esterases.

## Results and discussion

### Heterogeneous two-step synthesis in water

The starting CNF-t material was prepared by controlled TEMPO oxidation to achieve a high degree of oxidation, as already described by Isogai *et al.* in 2011.^7^ The first step of functionalization corresponds to a catalyzed amidation with furfurylamine performed in water using a large excess of both reactants and reagents. 1-Ethyl-3-(3-dimethylaminopropyl)carbodiimide (EDC) and *N*-hydroxysuccinimide (NHS) were used as coupling agents, and multiple washing steps were conducted in an effort to remove all the molecules that were not covalently bound, including the by-products issued from the coupling (EDC-urea, NHS) and excess of amine. Metronidazole was esterified with the 6-maleimido-hexanoic acid in the presence of *N*,*N*′-dicyclohexylcarbodiimide (DCC) as coupling agent, to give the metronidazole-maleimide precursor that was then used in the next step (see Fig. S1†).

Even if CNF-fur is supposed to be suitable for Diels–Alder click chemistry reactions with the prodrug molecule metronidazole-maleimide, the reaction efficiency is low considering the heterogeneous conditions and sparse furan groups. The reaction was therefore first implemented, validated and optimized by reacting furfurylamine with 6-maleimido-hexanoic acid in homogeneous phase (see Section 2 in ESI†) before being performed in similar conditions with CNF-fur and metronidazole-maleimide. Several washing steps were again performed to remove excess of reactants.

### Combining conventional characterization spectroscopy

The main goal was then to validate the different reaction steps, quantify the amount of functionalization present at each step, and finally to distinguish between adsorbed and covalently bound drug. Several experimental techniques (FTIR, solid-state NMR, *etc.*) were employed with the aim to gather information regarding the surface chemistry (see Section 4 in ESI†).

FTIR spectra of CNF-t, CNF-fur, and CNF-metro do not show noticeable changes (see Fig. S6†). The lack of sensitivity and resolution prevents any conclusion (even qualitative) to be made regarding the amount of drug loading and the presence of grafting *versus* adsorption. Solid-state NMR spectroscopy was also implemented to characterize surface species of the modified CNF. However, it was not possible to detect any of the reaction products, beyond the typical CNF core signals and the carbonyl signal resulting from this CNF oxidation. This suggests a rather small drug loading level. In the end, no conclusive answer regarding the mode of functionalization can be drawn from the combined use of the techniques mentioned above, which is consistent with previously published work.^16,26^ As a consequence, we then investigate whether dynamic nuclear polarization can be used to solve this limitation.

### DNP-enhanced solid-state NMR

**Fig. 2:**
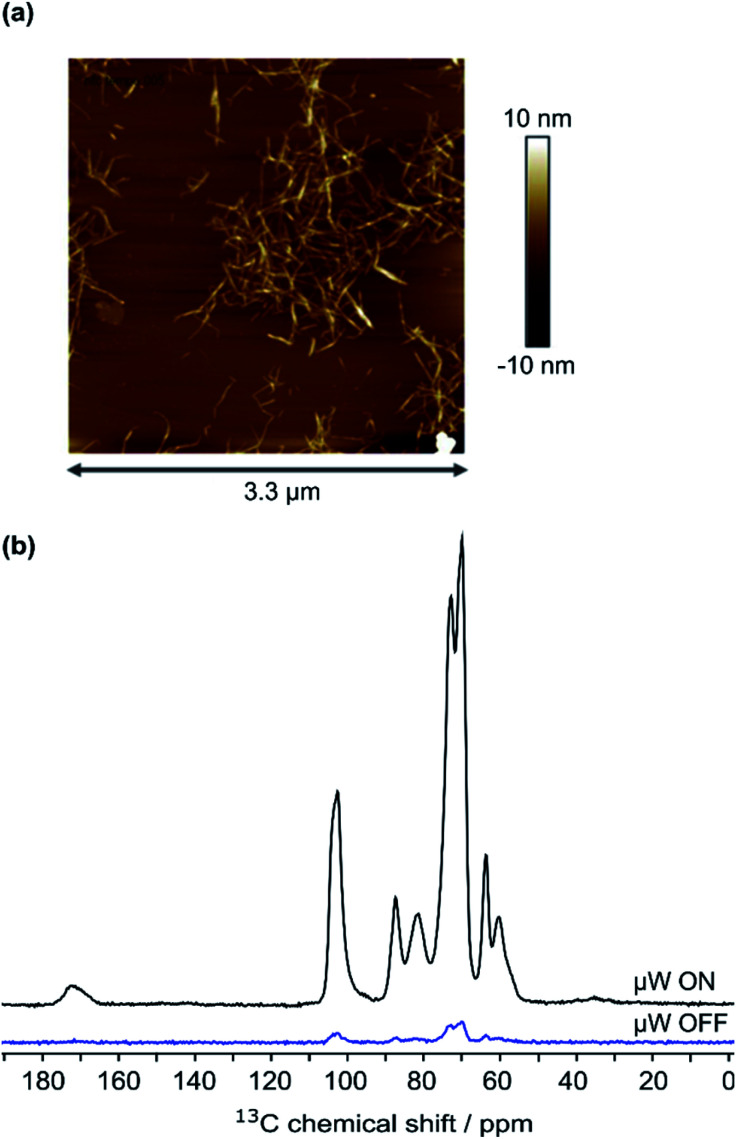
(a) AFM image of 7.5 × 10^−5^ wt% CNF suspension, (b) ^13^C CPMAS NMR spectra of CNF-metro with and without the application of microwave (μw) irradiation suitable for DNP.

**Fig. 3:**
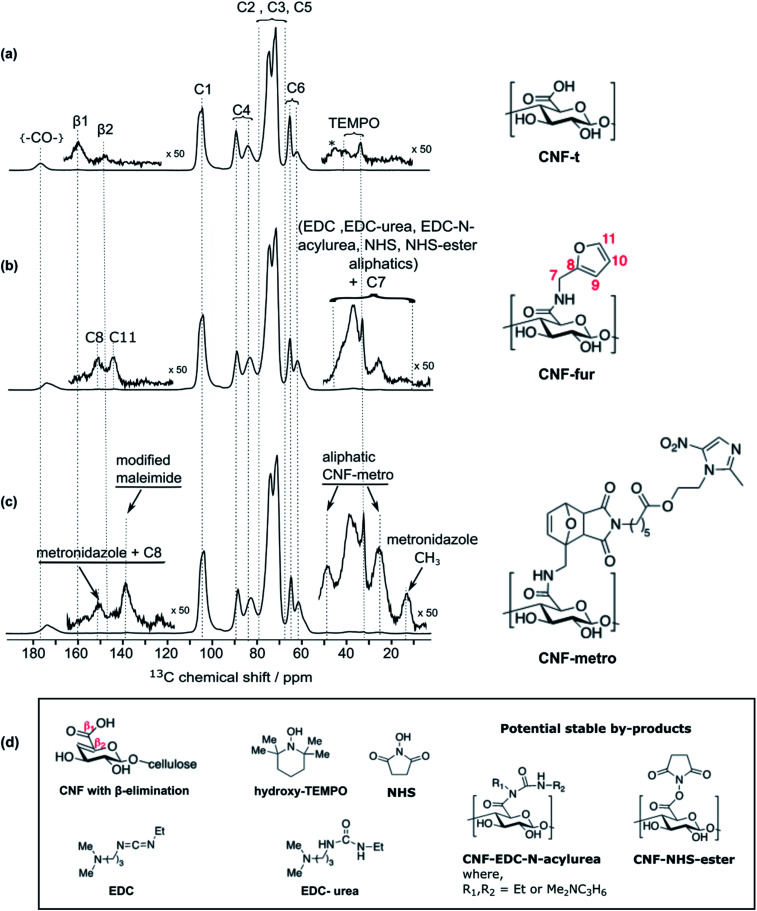
DNP-enhanced solid-state NMR of surface-modified CNFs: ^13^C CPMAS NMR spectra of (a) initial TEMPO-oxidized cellulose nanofibrils (CNF-t), (b) furylated cellulose nanofibrils (CNF-fur), and (c) maleimide-modified metronidazole on cellulose nanofibrils (CNF-metro). The cellulose ^13^C resonance assignment for (a–c) is given in the figure. The insets in (a–c) show magnified views of the 0–55 ppm and 115–165 ppm regions, with the corresponding ^13^C resonance assignment. (d) Chemical structures of CNF with β-alkoxy-elimination, hydroxyl TEMPO, the coupling agents EDC and NHS, and their potential stable by-products.

High-field dynamic nuclear polarization (DNP), an emerging hyperpolarization technique, is currently changing the scope of solid-state NMR spectroscopy since it allows enhancing the NMR sensitivity by several orders of magnitude.^27–29^ This technique relies on the use of optimized paramagnetic molecules,^30–33^ called polarizing agents, and suitable microwave irradiation to induce a significant increase of nuclear polarization. Recently, DNP-enhanced NMR has been shown as a powerful tool to understand surface chemistry of various systems^27,34–36^ including cellulose.^37,38^ In the experiments reported here, the abundant protons of the samples are hyperpolarized by DNP and ^1^H–^1^H spin diffusion at 100 K, typically in several seconds. The ^1^H hyperpolarization is then transferred to ^13^C or ^15^N using cross polarization. DNP experiments were performed on CNF-t, CNF-fur, and CNF-metro. In the three cases, large sensitivity gain was achieved compared to conventional NMR experiments (see Fig. 2b). The DNP-enhanced ^13^C Cross Polarization (CP) Magic Angle Spinning (MAS) NMR spectra of the different CNF samples are given in Fig. 3. In addition to the large resonances from the cellulose and the surface carboxylic groups that were already detected in standard solid-state NMR spectra, one can now observe additional signals in the aromatic and aliphatic regions. Also, it is worth noting that no aldehyde groups are observed in the ^13^C CPMAS NMR spectra of the CNF-t sample.

### Insight into the surface chemistry of TEMPO-oxidized CNFs: DNP enables the detection of traces of TEMPO moieties and β-alkoxy-elimination

Along with cellulose carbons (C1–C6) between 63 ppm to 105 ppm, the carbonyl groups resulting from TEMPO-mediated oxidation pre-treatment of cellulose can be seen at 177 ppm in the DNP-enhanced ^13^C CPMAS spectrum of the TEMPO-oxidised CNF (CNF-t) shown in Fig. 3a. The high-shifted carbonyl signal observed here is due to the alkaline conditions used (pH 10, NaOCl/NaOBr), which stabilize the negative charges on the CNF surface. This is confirmed by the presence of Na in the sample, as observed in DNP-enhanced ^23^Na CPMAS spectra (see Fig. S7†). This is thus fully consistent with the presence of sodium carboxylate groups at the CNF-t surface (stabilized due to pH 10). Fig. 3a also displays weak resonances between 10–50 ppm that can be assigned to the CH_2_ and methyl groups of reduced TEMPO moieties. Remaining resonances from the quaternary carbons in reduced TEMPO are expected around 65–68 ppm. Overlap with the much larger signal from the cellulose C6 resonance prevents their detection. Finally, the ^13^C DNP-enhanced NMR spectrum from Fig. 3a also shows weak resonances at 160 ppm and 148 ppm. Such resonances have never been reported so far and could only be observed for the CNF-t sample. These resonances are assigned to cellulose broken chains. Indeed, under alkaline conditions, it is well known that β-alkoxy-elimination of oxidized cellulose units can occur leading to 1 → 4 β-glycosidic bond cleavage.^7,39^ The corresponding ^13^C chemical shift were confirmed using the NMRdb online prediction tool.^40,41^

### Insight into the surface chemistry of CNF-t modified with furfurylamine: DNP enables the unambiguous detection of reacted coupling agents “invisible” with other techniques

**Fig. 4:**
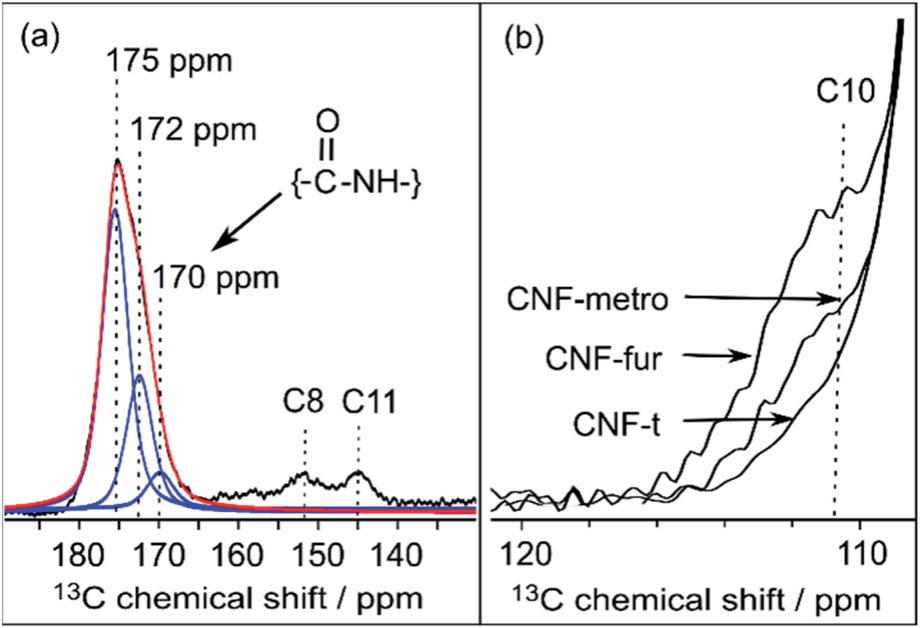
(a) Deconvolution of the signal between 170 and 180 ppm from CNF-fur sample, showing three distinct peaks (in blue) at 175, 172 and 170 ppm, which are respectively assigned to carboxylic function, carbonyl from coupling agents, and to amide carbonyl. The red line shows the result of the deconvolution (sum of the three contributions). (b) Extracted region between 120 and 110 ppm of the different ^13^C CPMAS NMR spectra of Fig. 3, highlighting the evolution of the C10 furan resonance at 111 ppm throughout the different samples.

In the DNP-enhanced ^13^C CPMAS NMR spectrum of CNF-fur displayed in Fig. 3b, two carbon resonances at 145 and 152 ppm are observed, which are assigned to furan C11 and C8, respectively. Resonances of the two remaining carbons, C9 and C10, from the furan ring are expected between 105–115 ppm. The C10 resonance can be observed as a shoulder on the left of the cellulose C1 resonance, as highlighted in Fig. 4b, while the furan C9 resonance overlaps with the cellulose C1 resonance and could not be observed. The C7 resonance is expected near 40 ppm both in the case of an amine (adsorption on CNF) or an amide function (covalent grafting onto CNF). In both cases, observation of this signal is hindered by the overlap with contributions from residual coupling agents (EDC, NHS) and/or reaction byproducts (EDC-urea, NHS-ester, *etc.*).^42^ It is thus not straightforward to differentiate between grafting and adsorption using the C7 resonance, especially since the peak intensities from the furan moieties are similar to the ones of the remaining coupling agents, suggesting a small amount of grafting.

At this point, it is importing to mention that intense purification methods, *i.e.* centrifugation–dispersion cycles and extensive dialysis, were employed to minimize the amount of remaining coupling agents. Nevertheless, DNP-enhanced NMR, as opposed to FTIR, UV and solid-state NMR, is still able to detect remaining traces.

### Differentiating grafting and adsorption

Even though the presence of furan is clearly seen in the DNP-enhanced spectra of CNF-fur, the crucial question of its mode of grafting, adsorbed on, or bound to the CNF surface, is not yet addressed. Answer to that question can be obtained through a detailed analysis of the broad CO resonance around 175 ppm in the DNP-enhanced CPMAS spectrum of CNF-fur (see Fig. 4a). Deconvolution of the asymmetric lineshape requires three components, whose chemical shifts were found at 175, 172, and 170 ppm. The most intense component at 175 ppm is close to the carboxyl chemical shift of CNF-t (177 ppm). It corresponds to unmodified carboxylic acid groups on the CNF surface. The difference in chemical shift (177 *vs.* 175 ppm) results from a change in pH between the two samples, as described above. The two other resonances at 170 and 172 ppm are more challenging to assign. The chemical shift at 170 ppm (the less intense component) is consistent with an amide resonance resulting from the covalent bonding of furfurylamine with the surface carboxyl. The corresponding signal intensity of this component matches the intensity of the other furan resonances (C8, C11). Tentative assignment of the last component at 172 ppm is less obvious, with possible contributions from adsorbed NHS, as well as stable grafted by-products such as EDC-*N*-acylurea and/or NHS-ester (see Fig. 3d).^42^

**Fig. 5:**
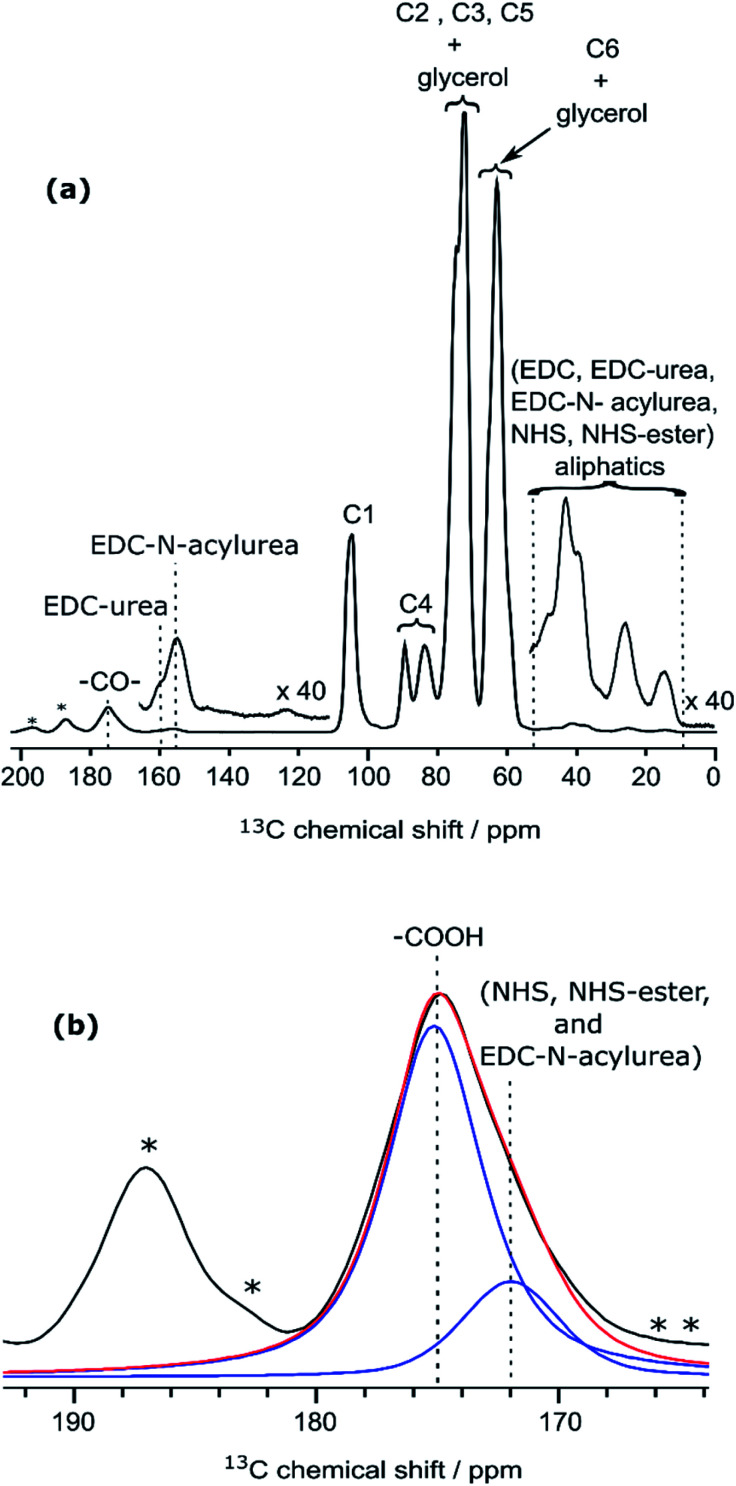
(a) DNP-enhanced ^13^C CPMAS NMR spectra of CNF-t modified in the presence of the coupling agents (EDC and NHS) only. Insets show magnified views of the 0–55 ppm and 110–165 ppm regions, with the corresponding ^13^C resonance assignment. Spinning sidebands are marked by asterisks. Note that glycerol was used in the DNP matrix. (b) Deconvolution of the carboxyl signal of (a), showing two contributions, at 175 and 172 ppm (in blue), corresponding respectively to carboxylic function and to carbonyl from coupling agents. The red line shows the result of the deconvolution (sum of the two contributions).The presence of EDC-*N*-acylurea is interesting as it has not been considered as a possible side-reaction of CNF-t functionalization. Indeed, amidation of CNF carboxyl groups generally proceed by their reaction with carbodiimides in the presence of NHS to prevent the formation of stable *N*-acylureas.^44^ Here we hypothesize that the kinetics of the NHS reaction is limited in the case of heterogeneous mixtures and can lead to the formation of *N*-acylureas.

To clarify this point, a new CNF sample was prepared using the same reaction conditions as for CNF-fur, but without adding furfurylamine. We refer to this sample as “CNF with coupling agents only”. The corresponding ^13^C DNP-enhanced CPMAS NMR spectrum is presented in Fig. 5a. Strikingly, deconvolution of the carbonyl signal in Fig. 5b reveals only a single additional contribution at 172 ppm (with similar relative intensity as in CNF-fur) next to the major resonance at 175 ppm, whereas the resonance at 170 ppm is totally absent. This confirms the resonance assignment of the 170 ppm peak to furfurylamide and of the resonance at 172 ppm to residual reacted and/or unreacted coupling agents on the CNF surface. We suggest here the formation of stable by-products (*N*-acylurea and NHS-ester) grafted on the CNF surface, as well as a potential contribution from adsorbed NHS.^42^ Resonances from aliphatic carbons of these reacted and unreacted coupling agents can be seen between 10–55 ppm, as shown in the inset of Fig. 5a. Furthermore, the presence of resonances at 156 and 159 ppm confirms the presence of EDC-*N*-acylurea and EDC-urea, in agreement with previous studies.^42,43^ The relative intensity of furan (C8 and C11) and amide (at 170 ppm) resonances (see Fig. 4a) can be used to claim that the amount of unreacted adsorbed furfurylamine is relatively low, or under the noise level.

### Diels–Alder reaction on furan-grafted CNFs

New resonances at 139, 49, and 14 ppm appearing in the DNP-enhanced CPMAS NMR spectrum of CNF-metro in Fig. 3c confirm the presence of maleimide-modified metronidazole. Furthermore, the decrease in intensity of the furan resonance at 110 ppm (see Fig. 4b) in the CNF-metro spectrum compared to the CNF-fur spectrum, and the presence of a new aromatic resonance at 139 ppm confirm the Diels–Alder reaction of the maleimide with the furan. The change in relative intensity of the resonance at 110 ppm gives an estimation for the yield of the Diels–Alder reaction of ∼50%, with respect to the number of surface furfuryl groups in CNF-fur.

### Quantifying surface species using DNP-enhanced NMR, from CNF-t to CNF-metro

DNP-enhanced CPMAS NMR spectra as analyzed so far provide a detailed qualitative picture of the surface species induced through the two-step reaction. They can however not be directly used to quantify these different species as CP spin-dynamics may vary for different types of carbon environments, in particular between protonated and quaternary carbon sites. In case of larger particles, the signal enhancement provided by DNP may also not be uniform between surface and bulk sites. This is however not the case here, as the size of the CNF used, about 10 nm in diameter, is sufficiently small to obtain a uniform DNP enhancement and mono-exponential hyperpolarization build-up times across the different resonances of the spectrum. To address the problem of non-uniform CP spin-dynamics, we implemented the quantitative MultiCP experiment,^45^ which was applied under DNP conditions to the CNF-t sample. Deconvolution of the C1, C4, C6 and CO signals leads to a precise integral calculation despite partial overlap of the different contributions (see Fig. S8 and Table S2†). For the quantitative analysis, the C4 integral was used as a reference (set to 100).

Our analysis reveals that the TEMPO oxidizing treatment induces a modification of the C6 carbon, resulting in ∼24% carboxylate groups in CNF-t (see Table S2†) with 74% of C6 carbons remaining unmodified. Carboxylate groups at 160 ppm resulting from β-alkoxy-elimination in the CNF-t sample can be estimated to ∼1.0% of the number of cellulose glucose units. Overall, the sum of the unmodified and oxidized C6 contributions is thus ∼99 ± 1%, which prove the self-consistency and accuracy of the analysis. Interestingly, no aldehydes are observed within our detection limit. Note that these groups could also appear as hemiacetals and acetals, whose weak ^13^C NMR signal would overlap with the intense C1 peak. The amount of remaining reduced TEMPO moieties is estimated to 0.5%.

Comparison of the relative integrals of C4 (or C1) and carbonyl resonances in CPMAS and MultiCP experiments of CNF-t allows the estimation of a CP-dynamics correction factor, which can then be used to further quantitatively analyse the DNP-enhanced ^13^C CPMAS NMR spectra of CNF-fur and CNF-metro. Thus, the degree of furfurylamine substitution in CNF-fur can be estimated to ∼2% compared to a glucose unit of cellulose based on the 170 ppm peak intensity. Comparatively, the peak at 172 ppm, assigned to coupling agent derivatives, contributes to about 7%. It is important to note that most of these correspond to compounds that have reacted with the CNF surface carboxylic groups, forming stable species (that cannot be washed away), such as *N*-acylurea from EDC and NHS-ester from NHS, but also to adsorbed NHS moieties.^42,43^

Finally, the well-isolated metronidazole methyl resonance present in the CNF-metro DNP-enhanced ^13^C CPMAS NMR spectrum can be used to roughly estimate the degree of drug grafting to ∼1% compared to the glucose unit in cellulose. This is consistent with the amount of unreacted furfuryl in CNF-metro (estimated to ∼1% using the peak at 145 ppm) and the estimated yield of ∼50% for the Diels–Alder reaction step.

Table 1 summarizes the amount of surface species revealed by DNP-enhanced solid-state NMR spectroscopy, relative to the cellulose C4 peak for CNF-t, CNF-fur, and CNF-metro. More details can be found in ESI Section 5.†

**Table 1:** Amount of surface species in % of glucose units obtained by DNP-enhanced NMR^a^

Signal integral	CNF-t	CNF-fur	CNF-metro
C4	100	100	100
C6 unmodified	74 ± 1	74 ± 1	74 ± 1
All CO signals	25 ± 1	27 ± 1	30 ± 1
CO signals from coupling agent	—	7 ± 1	7 ± 1
C6 COOH	24 ± 1	18 ± 1	18 ± 1
Grafted furfurylamine	—	2 ± 0.2	2 ± 0.2
Adsorbed furfurylamine	—	<0.2	<0.2
Grafted drug	—	—	1 ± 0.2
Adsorbed drug	—	—	<0.2

a Errors on the relative integrals of isolated peaks have been estimated to 0.2% of the C4 resonance, based on the noise level of the experiments. Errors on integrals obtained from deconvolution are higher and estimated to be about 1%.

### Atomic-scale information *versus* global information on surface modification – comparison with standard analytic tools

The discussion above demonstrates that precise and quantitative atomic-scale information can be gathered on low-level CNF surface modification. Such information is of tremendous importance to further develop green synthetic routes for heterogeneous CNF surface reactions, and is currently beyond reach for other spectroscopies. Nevertheless, much work dealing with CNF surface modification reports the use of elemental analysis and conductometric titration to prove and estimate surface loading. In the case of elemental analysis, it has been reported that the sensitivity can be sufficient to detect changes in wt% of carbon, nitrogen and sulfur (if present) content.^20,46^ This can be used to confirm the presence of sulfur groups after acid hydrolysis^46^ and also to check surface modifications through the appearance of nitrogen or sulfur. Changes in proton, oxygen and carbon mass fractions are much more difficult to use because of the presence of contaminant and residual adsorbed water. As for conductometric titration, the degree of oxidation (DO) can be calculated for both unmodified and modified CNF samples. The difference in the DO value between the two samples is often used to estimate the amount of drug directly bound to CNF.^20,21,26^ Elemental analysis and conductometric titration were thus implemented on the three CNF samples (see Sections 4.1 and 4.3 in ESI†) with the goal to compare the results with those obtained with DNP-enhanced solid-state NMR spectroscopy.

Concerning the results of the elemental analysis on CNF-t (see Table S1†), the first thing to note is that the mass proportion of C, O, and H does not match with the composition of cellulose carrying any amount of surface carboxylate/carboxylic acid groups, with the carbon proportion being too low compared to the measured mass of H and O. This mismatch can probably be explained by the presence of adsorbed water molecules, but also by the results obtained with DNP-enhanced NMR, *i.e.* the presence of residual reduced TEMPO moieties as well as of β-alkoxy-elimination derivatives. Additionally, the presence of contaminants during the production process cannot be ruled out. From CNF-t to CNF-fur, the elemental analysis reports an increase in nitrogen and carbon mass proportion of ∼0.47% and ∼4%, respectively, while the oxygen wt% is decreased by ∼0.6%. These results are not consistent with the furfurylamine functionalization alone since an increase in nitrogen mass proportion of 0.47% should correspond to an increase of 1.9 and 0.5% in carbon and oxygen wt%, respectively. As a comparison, the DNP-enhanced NMR analysis estimates the amount of furfurylamine functionalization to be ∼2% with respect to glucose units in cellulose. This would translate into an increase of 0.2% and 0.9% in nitrogen and carbon wt%, respectively, which is much lower than the values observed by elemental analysis.

Conductometric titration estimates the number of glucose units bearing a carboxyl group in CNF-t to be ∼30.1%, while the DNP-enhanced NMR analysis gives ∼25%. This difference can partly be explained by the presence of remaining TEMPO moieties as well as other residual contaminants, which cannot be ruled out. Similarly to elemental analysis, conductometric titration overestimates the level of furfurylamine grafting as well. The analysis yields ∼12% of furan grafting as compared to ∼2% obtained by DNP-enhanced NMR. This difference can be largely explained by the presence of residual coupling agents bonded to CNF carboxylic acid groups (*N*-acylurea and NHS ester) and remaining reduced TEMPO moieties. This has so far been overlooked in the interpretation of conductometric titration analysis.

## Conclusions

It has been shown that the large NMR sensitivity gains achieved by DNP enables the understanding of the surface chemistry of CNFs with unprecedented detail and the unambiguous characterization at natural isotopic abundance of low weight percentage of grafted API. Here, the API has on-demand release capability in the presence of esterases thanks to the design of a smart drug carrier formulation. The drug bonded to the CNF surface is estimated to about 1% of the glucose units of cellulose. Moreover, we show that DNP-enhanced NMR is the only technique that allows differentiating between grafting and adsorption in this case. Importantly, DNP-enhanced NMR spectroscopy also reveals the presence of other surface species, such as coupling agents (EDC/NHS, *etc.*), reduced TEMPO moieties, as well as the presence of β-alkoxy-elimination products. The presence of such organic moieties clearly impedes the reliable use of techniques such as elemental analysis and conductometric titration, which provide an overestimation of the drug loading without any means to differentiate between grafting and adsorption. This work should prove very useful to advance not only the field of cellulose-based smart drug carriers but also the full characterization of CNF-based materials.

## Experimental section

### Materials

4-(Dimethylamino)pyridine (DMAP, CAS: 1122-58-3), *N*,*N*-dicyclohexylcarbodiimide (DCC, CAS: 538-75-0), trifluoroacetic acid (TFA, CAS: 76-05-1), metronidazole (CAS: 443-48-1), furfurylamine (CAS: 617-89-0), *N*-(3-dimethylaminopropyl)-*N*′-ethylcarbodiimide hydrochloride (EDC, CAS: 25952-53-8), *N*-hydroxysuccinimide (NHS, CAS: 6066-82-6), sodium hydroxide (NaOH, CAS: 1310-73-2), hydrogen chloride (HCl, CAS: 7647-01-0) were purchased from Sigma Aldrich, Alfa Aesar or Acros Organics and used without further purification. Solution NMR spectra were recorded at room temperature in 5 mm tubes on a Bruker AC 400 MHz spectrometer (NMR facility, PCN-ICMG, Grenoble). Chemical shifts (*δ*) are reported in parts per million (ppm) from low to high field and referenced to residual non-deuterated solvent relative to Me_4_Si. Standard abbreviations for multiplicity were used as follows: s = singlet; d = doublet; t = triplet; m = multiplet. High resolution mass spectrometry (HRMS) was carried out on a Waters Xevo G2-S-QTof mass spectrometer using ElectroSpray Ionisation (NMR facility, PCN-ICMG, Grenoble).

### Synthesis of metronidazole-maleimide (Metro-MAL)

6-Maleimido-hexanoic acid^47,48^ (753 mg, 3.6 mmol), metronidazole (**2**, 626 mg, 3.6 mmol) and DMAP (40 mg, 0.36 mmol) were dissolved in CH_2_Cl_2_ (25 ml) at 0 °C. DCC (905 mg, 4.4 mmol) was added after 15 minutes. The reacting mixture was stirred for 4 h at room temperature (rt), then filtered and concentrated under vacuum. The crude product was purified by flash chromatography on silica gel with CH_2_Cl_2_/MeOH 98/2 (v/v) as eluent to give the compound as a yellow amorphous solid (669 mg, 1.83 mmol, 51%). ^1^H NMR (CDCl_3_, 400 MHz) *δ* 7.96, (s, 1H), 6.69 (s, 2H), 4.59 (t, 2H, *J* = 5.3 Hz), 4.41 (t, 2H, *J* = 5.3 Hz), 3.50 (t, 2H, *J* = 7.2 Hz), 2.51 (s, 3H), 2.26 (t, 2H, *J* = 7.5 Hz), 1.63–1.54 (m, 4H), 1.31–1.25 (m, 2H); ^13^C NMR (CDCl_3_, 100 MHz) *δ* 172.7, 170.8, 150.8, 134.1, 133.2, 62.4, 45.1, 37.5, 33.7, 28.2, 26.1, 24.1, 14.4; HRMS (ESI) *m*/*z*: calc. for C_16_H_21_N_4_O_6_ [M + H]^+^ 365.1461, obs. 365.1468. More details can be found in ESI Section 1.†

### TEMPO-oxidation of cellulose nanofibrils (CNF-t)

The cellulose nanofibril (CNF) suspension was provided by the Centre Technique du Papier (CTP, Grenoble, France). Suspension of TEMPO-oxidized CNF (CNF-t) was produced from a pre-refined (40° SR) bleached bisulfite pulp provided by Rayonier Advanced Materials (previously TEMBEC). The pulp concentration was adjusted to 1.5 wt% and the oxidation was performed at pH 10 for 2 h in the presence of NaBr, NaClO, and the TEMPO reagent. A high pressure homogenizer from GEA (Niro, Soavi, Italy) was used to defibrillate the oxidized cellulose fibers and produce the CNF-t suspension. The produced CNF-t suspension is in the form of a thick opaque gel at 1.6 wt%. AFM images of the low concentrated CNF-t suspension confirmed the nanosized morphology of CNF-t (see Fig. 2a). Using conductometric titration (CT), the degree of oxidation (DO) of the CNF-t suspension was found to be 30.1%, (see Fig. S5†).

### Functionalization of CNF-t with furan (CNF-fur)

The CNF-t suspension concentration was decreased from 1.5 wt% to 0.4 wt% in order to be easily stirred. Deionized water was added before homogenization with an IKA Ultra-Turrax high shear mixer for 1 min at 10 000 rpm. The pH of the suspension was then adjusted to 4 under magnetic stirring using a 0.5 M HCl solution.

A solution of the coupling agents EDC and NHS was prepared in deionized water and added to the suspension of CNF-t with a molar ratio of 4 equivalents of EDC and NHS for 1 equivalent of CNF-t surface carboxyl group (using the 30.1% DO observed by CT). The mixture was stirred for 30 min at rt at pH 4 and then the pH was increased to 8.5 before amine addition. Furfurylamine, with a molar ratio of 4 equivalents of amine for 1 equivalent of carboxylic acid of CNF-t, was mixed with 5 ml of deionized water and added to the mixture that was then stirred at rt with the pH maintained at 8.5.

After 72 h of reaction, the reaction was quenched by decreasing the pH to 2–2.5 with 0.5 M HCl solution. To remove non-covalently bound chemicals (EDC, NHS and free amine), 6 cycles of washing, centrifugation (10 min at 11 100 rpm) and re-dispersion with a high shear mixer (Ultra-turrax, IKA) for 1 min at rt were repeated. The last dispersion was done in neutral water to afford the furan modified CNF-t, referred to as CNF-fur.

The last step of purification consisted in a dialysis of the CNF-fur suspension against neutral water with 6–8 kDa MWCO membranes (Spectra/Por® 1 Standard RC Tubing, SPECTRUM) for at least 5 days under slow magnetic stirring and renewal of the medium twice a day.

### Diels–Alder reaction of Metro-MAL on CNF-fur

The Diels–Alder reaction was first optimized under homogeneous conditions before being implemented on the CNF-fur sample (see ESI Section 2†). Metro-MAL (95.2 mg) was dissolved in 40 ml of 1 : 1 mixture of deionized water and ethanol (v/v) in an ultrasound bath (IKA, USA). The CNF-fur suspension was diluted to 0.15 wt% concentration and the Metro-MAL solution (9.42 ml) was added dropwise under magnetic stirring in order to reach one molar equivalent of Metro-MAL to the furan groups available on the CNF substrate. The Diels–Alder reaction was then triggered by heating the system at 40 °C for 24 h under continuous stirring. The reaction was followed with UV analysis. The reacted CNF-fur, now referred to as CNF-metro, was washed with several centrifugation/re-dispersion cycles as described above to remove remaining reactants. The first 3 cycles were done with 1 : 1 (v/v) water/ethanol solution, followed by 2 cycles using deionized water only. Washed suspension was further purified by dialysis (6–8 kDa MWCO membranes, Spectra/Por® 1 Standard RC Tubing, SPECTRUM) against deionized water under slow magnetic stirring for 5 days with daily renewal of the dialysis medium. CNF-metro suspension was stored at 5 °C.

### AFM, FTIR, conductometric titration, and elemental analysis of CNF samples

Characterization of the different CNF (CNF-t, CNF-fur and CNF-metro) were performed on thin films and/or cryogel powders. Thin films were produced by solvent casting in Petri dishes of 0.1 wt% CNF suspensions in order to obtain 30 g m^−2^ films. The films were dried overnight in an oven at 40 °C. An alternative dry form of the different CNFs was obtained by freeze drying the suspensions (cryogel). The results obtained with AFM, conductometric titration, FTIR and elemental analysis can be found in ESI Sections 3 and 4.

### DNP-enhanced solid-state NMR

DNP-enhanced NMR experiments were performed on the three CNF samples (CNF-t, CNF-fur and CNF-metro). The samples were prepared by impregnating the CNF powder with a DNP matrix (1) composed of 10 mM AMUPol^30^ in D_2_O. For CNF-t, 28 mg (aerogel) was impregnated with 40 μL of the DNP matrix (1). For CNF-fur and CNF-metro, 30 mg of each were impregnated with 80 μL of DNP matrix (1), an additional CNF sample was prepared following the synthesis used for CNF-fur, except that no furfurylamine was added. We refer to this sample as the CNF sample with coupling agents only. In this case, a DNP matrix (2) of 10 mM AsymPolPOK^31^ in glycerol/D_2_O/H_2_O (60 : 30 : 10; v/v) was used. 25 mg of CNF with coupling agents only was impregnated with 50 μL of DNP matrix (2). Each sample was then fully packed into a 3.2 mm outer-diameter sapphire rotor.

All experiments were performed on a Bruker Avance III 400 MHz (^1^H resonance) DNP-NMR spectrometer equipped with 263 GHz gyrotron for microwave irradiation, a corrugated transmission line, and a low temperature 3.2 mm MAS probe used in double-resonance mode.^49^ All experiments were performed at a sample temperature of 100 K, using a magic-angle spinning (MAS) frequency of 13.3 kHz. Cross-polarization under MAS^50^ (CPMAS) experiments were performed with a radio frequency (rf) field strength of 50 kHz on the ^13^C while ^1^H rf field strengths were adjusted accordingly depending upon the ramp^51^ used to match CP conditions. The CP decay time constants were measured and were found to be in excess of 25 ms. The CP contact time was set to 2 ms for CPMAS experiments and 1 ms for the quantitative CP experiment using the Multi-CP^45^ pulse sequence. A 50-to-100% and 90-to-100% ramp were used for the ^1^H CP spin-lock in the CPMAS and quantitative CP using the MultiCP, respectively.^51,52^ The inter-scan delay was optimized according to the hyperpolarization build-up time of each sample, and set to 3.0 s for CNF-t, 2.5 s for CNF-fur, 1.9 s for CNF-metro and 1.9 s for CNF with coupling agents only. For the MultiCP experiment the inter-scan delay and the delay between each CP block were set to 3.9 s and 6 s, respectively. All experiments were processed and analyzed using Bruker Topspin 3.2 software, including the peak deconvolution and integration carried out to perform the analysis herein. ^13^C peak intensities are discussed relatively to carbon magnetization from the glucose unit of cellulose. The signal-to-noise of DNP-enhanced ^13^C CPMAS NMR spectra is about 500 : 1 for each of the carbon resonances of the glucose unit.

## Abbreviation

FTIR: Fourier transform infra-red
NMR: Nuclear magnetic resonance
DNP: Dynamic nuclear polarization
CNFs: Cellulose nanofibrils

## Conflicts of interest

There are no conflicts to declare.

## Supplementary Material

SC-011-C9SC06312A-s001
