# Supplementary material for: The surface chemistry of a nanocellulose drug carrier unravelled by MAS-DNP
Source: Chem Sci. 2020 Mar 13;11(15):3868–77. doi: 10.1039/c9sc06312a (PMC8152408; doi:10.1039/c9sc06312a)
Supplement: SC-011-C9SC06312A-s001 [file SC-011-C9SC06312A-s001.pdf]

Electronic Supplementary Material (ESI) for Chemical Science.
This journal is © The Royal Society of Chemistry 2020

SUPPLEMENTARY INFORMATION

The surface chemistry of nanocellulose drug carrier unravelled by MAS-DNP

Akshay Kumar,‡[a] Hippolyte Durand,‡[b] Elisa Zeno,[c] Cyril Balsollier,[d,e] Bastien Watbled,[e] Cecile Sillard,[b] Sébastien Fort,[d] Isabelle Baussanne,[e] Naceur Belgacem,[b] Daniel Lee,[a] Sabine Hediger,[a] Martine Demeunynck,[e] Julien Bras*[b] and Gaël De Paëpe*[a]

[a] Univ. Grenoble Alpes, CEA, CNRS, IRIG-MEM, Grenoble, France.
[b] Univ. Grenoble Alpes, CNRS, Grenoble-INP, LGP2, Grenoble, France.
[c] Centre Technique du Papier (CTP), Grenoble, France.
[d] Univ. Grenoble Alpes, CNRS, CERMAV, Grenoble, France.
[e] Univ. Grenoble Alpes, CNRS, DPM, Grenoble, France.

INDEX:

1. NMR spectra of the metronidazole-maleimide

2. Optimization of the Diels-Alder reaction on model compound

3. AFM on TEMPO-oxidized CNF

4. Characterization and quantification of chemical modifications

4.1. Conductometric titration

4.2. FTIR spectroscopy

4.3. Elemental analysis

5. DNP-enhanced solid-state NMR spectroscopy

1. NMR SPECTRA OF METRONIDAZOLE-MALEIMIDE

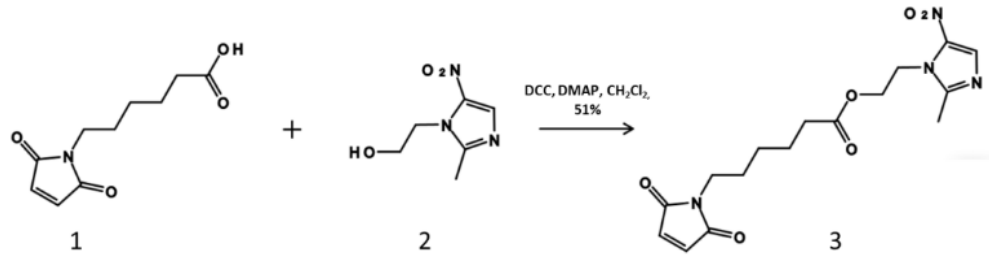

Fig. S1 Synthesis pathway of metronidazole-maleimide (3) from metronidazole (2) and 6-maleimido-hexanoic acid (1).

Liquid-state NMR spectra were recorded at room temperature in 5 mm tubes on a Bruker AC 400 MHz spectrometer (NMR facility, PCN-ICMG, Grenoble). Chemical shifts (δ) are reported in parts per million (ppm) from low to high field and referenced to residual non-deuterated solvent relative to TMS.

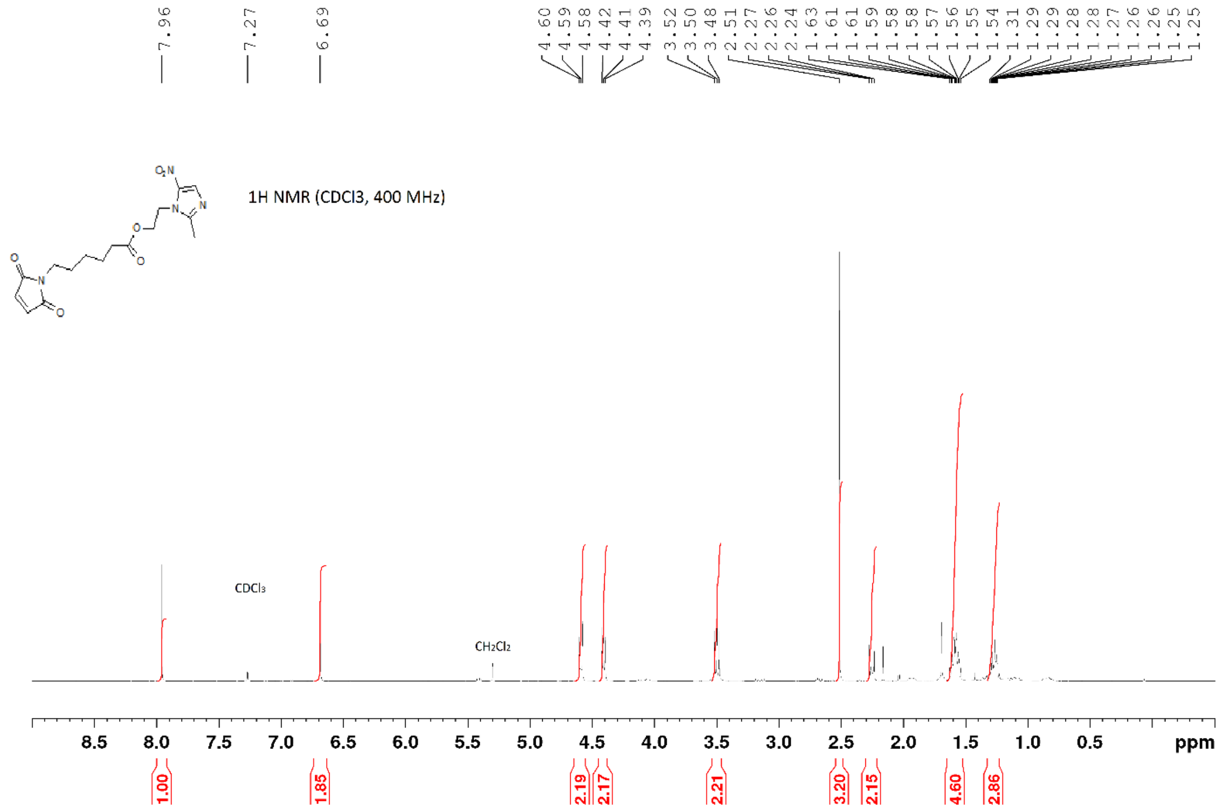

Fig. S2 Liquid-state ^{1}H NMR spectrum of metronidazole maleimide in $CDCl_3$.

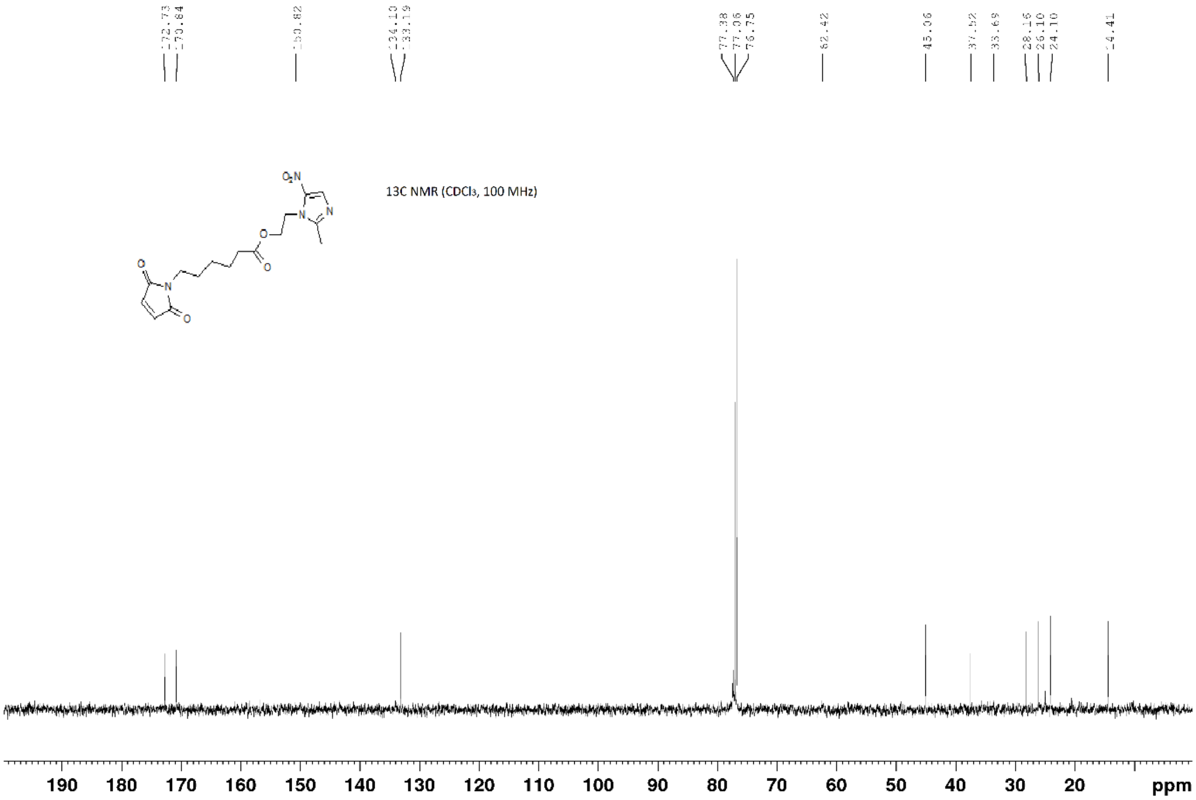

Fig. S3 Liquid-state ^{13}C NMR spectrum of metronidazole maleimide. The $CDCl_3$ solvent peak appears at 77 ppm.

The chemical structure of the Metro-MAL compound (metronidazole maleimide) was confirmed on the base of its ^{1}H and ^{13}C liquid-state NMR spectra (Fig. S2 and S3). The prodrug chemical structure is suitable for the Diels-Alder reaction since the maleimide group is readily available on the isolated molecule.

2. OPTIMIZATION OF THE DIELS-ALDER REACTION ON MODEL COMPOUND

Furfurylamine (Fur) and 6-maleimido-hexanoic acid (6-MHA) were mixed in an equimolar ratio in a mixture of EtOH-H_2O (50/50 v/v). The solution was maintained at 40°C and the pH was kept at 6 (± 0.05). Chromium (III) acetylacetonate relaxing agent was added in 10 mg/ml final concentration. The experiment was repeated in an NMR tube to record spectra over time in order to assess the kinetics of the model Diels-Alder reaction. Spectra were recorded at 15 min, 1 h, 5 h, 24 h, 48 h and 72 h, and are displayed in Fig. S4.

The liquid-state ^{13}C NMR spectra were recorded at the Laboratoire Modélisation et Exploration des Matériaux (MEM) in the «French Alternative Energies and Atomic Energy Commission (CEA) » at Grenoble, on a Bruker AVANCE400 spectrometer. Acquisition and data processing was done using the Bruker TopSpin 3.2 software. The experiments were recorded with a relaxation delay of 5 s. Chemical shifts are given relative to TMS (tetramethylsilane, $\delta = 0$ ppm). The positions of the peaks were referenced to the residual solvent signal. To discriminate unambiguously the -CH / -CH_3 from the -CH_2, the Bruker *DEPT* sequence was used with 0.648 s acquisition time, 3.0 s relaxation delay, a last pulse at 135° which selects CH_2 carbons with a reverse sign compared to CH and CH_3. A coupling constant of 145 Hz was considered.

The diene carbons involved in the Diels-Alder cycloaddition give rise to new signals after the reaction since they belong to the carbons that undergo the strongest modification of chemical environment. The evolution of the liquid-state ^{13}C NMR spectra during the reaction is displayed in Fig. S4 for the 79 to 92 ppm chemical shift region, together with the chemical compounds involved in the reaction. This chemical shift region reveals the apparition of characteristic peaks that indicate the success of the Diels-Alder reaction. After 1 h of reaction at 40°C different peaks start to appear and to get stronger and more defined after 5 h and 24 h. These signals are not increasing anymore after 48 h. The signal at 88 ppm and the multiplet centered at 91 ppm are associated with the C10 carbon of the reaction product, while the peaks at 79 and 81 ppm correspond to the C13 carbon. The apparition of these signals confirms that the Diels-Alder reaction is occurring under the selected conditions in homogeneous phase. Even if it was expected that the reaction kinetics is slower under heterogeneous conditions, this experiments gave us a rough idea of the time required to activate the Diels-Alder reaction. Based on these test results, we decided to perform the Diels-Alder reaction on CNF-fur for at least 24 h.

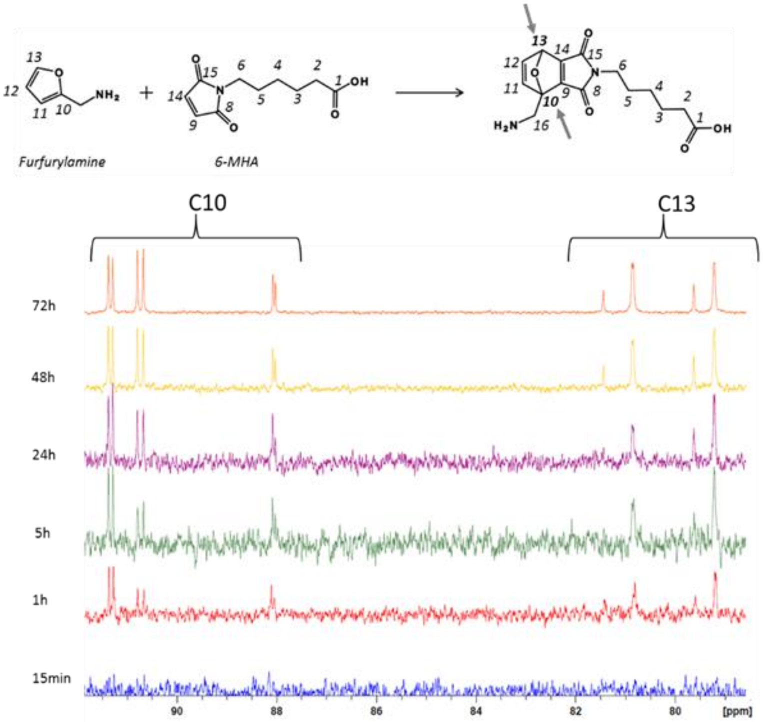

Fig. S4 Evolution of the liquid-state ^{13}C NMR spectra of the reaction mixture of furfurylamine and 6-maleimido-hexanoic acid in a 50/50 v/v EtOH/deionized D_2O solvent showing the apparition of the Diels-Alder reaction product as a function of time. Only the 79-92 ppm chemical shift region is displayed.

3. AFM ON TEMPO-OXIDIZED CNF

Properties and characteristics of CNF can differ from one supplier to another. The CNF-t suspension at 1.6 wt% concentration was in the form of a thick gel. AFM images of a low concentrated CNF-t suspension were recorded to confirm the nano-size morphology of the CNF (see Fig. 2). They were obtained on a Dimension icon® (Bruker) on a 7.5×10^{-5} wt% suspension prepared by iterative dilutions of the gel using a high shear mixer Ultra-Turrax (IKA) to keep a good homogeneity even at low concentration. A drop of this suspension was deposited on freshly cleaved mica plate before drying overnight under the fume hood at rt. The acquisition was performed in tapping mode using a silica coated cantilever (OTESPA® 300 kHz – 42 N/m, Bruker, USA). Zones of 3.3*3.3 μm^2 were analyzed. At least five images on two different samples were performed and the most representative ones were selected for the discussion.

4. CHARACTERIZATION AND QUANTIFICATION OF CHEMICAL MODIFICATIONS

Conductometric titration, FTIR spectroscopy, and elemental analysis are three techniques routinely used to evidence and quantify chemical modifications of CNF surfaces. We describe below the methodologies used in this work.

4.1 CONDUCTOMETRIC TITRATION

Conductometric titration was performed on CNF-t and CNF-Fur to estimate the content of carboxylic moieties before and after the first step of functionalization. 50 mg of dry CNF-t was precisely suspended in 200 ml of de-ionized water. Magnetic stirring and high shear mixer Ultra-Turrax (IKA) were used to produce a homogeneous dispersion. The pH was then decreased around 2.5 using 0.1 M HCl to protonate all carboxylate groups at the CNF surface and the volume of added HCl solution was recorded. The titration was then performed using a 0.01 M NaOH solution. The conductivity of the suspension was recorded stepwise, waiting for stabilization at each step, as a function of the added NaOH volume. Smaller steps (i.e. smaller volume of added NaOH) were used where necessary to better reproduce large slope changes. All titrations were repeated at least three times.

The titration curves typically display three regions, a first slope showing the decrease of the conductivity that corresponds to the neutralization of the remaining strong acid, a plateau where the weak acid groups are titrated (carboxylic groups of CNFs) and a last part where the conductivity increases when all acids are titrated. The degree of oxidation (DO) represents the number of carboxylic groups per anhydroglucose unit (AGU) on CNF-t. It can be calculated with equation (1) following Da Silva Perez *et al.*[1]:

$$DO = \frac{162 \times C \times (V_{eq1} - V_{eq2})}{m - 36 \times C \times (V_{eq1} - V_{eq2})}. \quad (1)$$

The value of 162 (in $g \cdot mol^{-1}$) in the equation is the molar mass of the AGU; C (in $mol \cdot l^{-1}$) is the concentration of the NaOH solution; m (in g) is the mass of dry CNF-t; the value of 36 (in $g \cdot mol^{-1}$) corresponds to the difference between the molar mass of the carboxylate form of the carboxylic groups including the sodium counter ion (198 $g \cdot mol^{-1}$) and the molar mass of the AGU (162 $g \cdot mol^{-1}$). This term arises from the partial replacement of primary hydroxyl groups by carboxylate groups during the TEMPO mediated oxidation process. The two NaOH solution equivalent volumes V_{eq1} and V_{eq2} are extracted at the edge of the plateau on the titration curve by calculating the intersections of the first (decreasing slope) and the third (increasing slope) part of the curve with the plateau.

The carboxylic group content (X_{ox}) on CNF-t can be calculated with equation (2):

$$X_{ox} = \frac{C \times (V_{eq1} - V_{eq2})}{m}. \quad (2)$$

X_{ox} is expressed in micro-mole of carboxylic group per gram of dry CNF ($\mu mol \cdot g^{-1}$).

After the amidation reaction with furfurylamine, the amount of remaining carboxylic group can also be assessed by conductometric titration. The residual degree of oxidation (DO_{res}) is calculated with equation (3) by integrating the molecular weight M of the furfurylamine and the same previous terms as it was done before for amidation on CNF:[2]

$$DO_{res} = \frac{(162 + (M-4) \times DO) \times C \times (V_{eq1} - V_{eq2})}{m - (M-40) \times C \times (V_{eq1} - V_{eq2})}. \quad (3)$$

Equations (1) and (3) are used to compare the DO before and after the amidation reaction. The proportion of carboxylic groups (% $COOH_{conv}$) that were converted during the amidation reaction can thus be assessed with equation (4):

$$\% \, COOH_{conv} = \frac{DO - DO_{res}}{DO} \times 100 \, . \quad (4)$$

From the values of X_{ox} and % $COOH_{conv}$ it is possible to calculate the degree of substitution, DS_{cond}, based on conductometric titration measurements. By considering one gram of CNF-t, X_{ox} gives access to the molar quantity of the oxidized AGU before amidation. Non-oxidized AGU (n [AGU-OH]) can be determined by subtraction as given in the equation (5.2). Through amidation of CNF-t, only oxidized AGU will react and % $COOH_{conv}$ gives access to the molar quantity of converted oxidized AGU (n [AGU-FUR]) and non-reacted oxidized AGU (n [AGU-$COOH_{nr}$]) by subtraction again (see equation (5.3)). Equation (5) gives the degree of substitution:

$$DS_{cond} = \frac{n[AGU-FUR]}{n[AGU-OH]+n[AGU-COOH_{nr}]+n[AGU-FUR]} \, , \quad (5)$$

Where

$$n[AGU - FUR] = \%COOH_{conv} \times X_{ox} \, , \quad (5.1)$$

$$n[AGU - OH] = \frac{1 - X_{ox} \times M_{AGU-COOH}}{M_{AGU-OH}} \, , \quad (5.2)$$

$$n[AGU - COOH_{nr}] = 1 \times X_{ox} - n[AGU - FUR] \, . \quad (5.3)$$

$M_{AGU-COOH}$ and M_{AGU-OH} are the molar masses of an oxidized AGU (176.1 g·mol^{-1}) and an AGU (162.1 g·mol^{-1}), respectively. In equation (5.3), the value of 1 (in g) is a mass, as one gram of modified CNF is considered for the DS_{cond} calculation.

Conductometric titrations were performed on the CNF-t suspension to assess the degree of oxidation DO and initial carboxylic acid group content X_{ox} that describe the chemical reactivity of the material. A DO around 30.1 % and a X_{ox} of 1.7 ± 0.1 mmol/g were calculated, which confirmed the high surface charge and surface chemical reactivity introduced on CNF surface through the TEMPO mediated oxidation treatment.

Conversion of carboxylic acids of CNF-t to any other carbonyl function was confirmed by conductometric titrations performed after the amidation and purification steps. The objective was to assess the amount of unreacted carboxylic acid groups and calculate the residual degree of oxidation DO_{res}. Fig. S5 shows the most representative curves obtained and the values of DO and DO_{res} obtained before and after the reaction, calculated with equations (1) and (3), respectively. The DO_{res} measured for CNF-fur is found to be 18.3 % compared to 30.1 % for initial CNF-t suspension. The proportion of converted carboxylic group % $COOH_{conv}$ can thus be calculated with equation (4) and gives a value of 39 %, which can further be translated into a degree of substitution DS_{cond} = 0.11. However, it is to note that the conductometric titration for this type of systems cannot distinguish between the different types of carbonyl functions built upon reaction, for instance between bounded furfurylamine and reacted coupling agents on CNF.

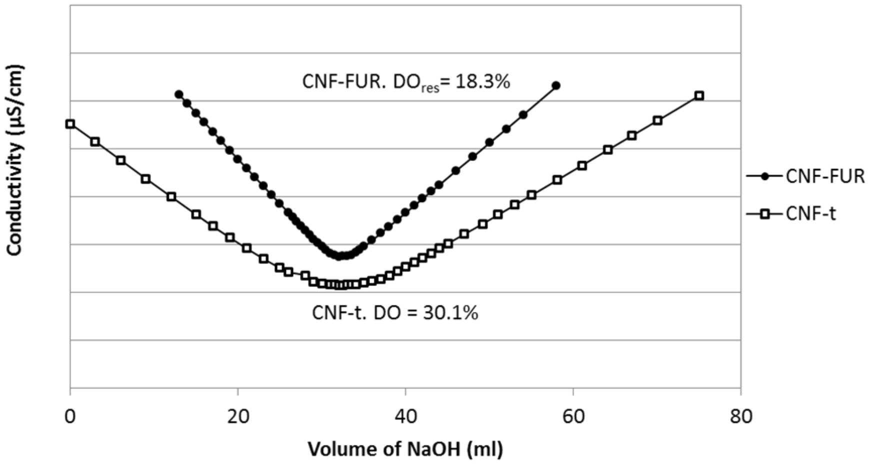

Fig. S5 Conductometric titration of CNF-t and CNF-fur suspension.

4.2. FTIR SPECTROSCOPY

Spectra of the films were obtained on a Perkin Elmer Spectrum One spectrometer (Waltham, Massachusetts, USA). KBr pellets were prepared with one drop of CNF suspensions to analyze. For each sample (3 pellets per sample), measurements of 16 scans with a resolution of 2 cm^{-1} between 600 cm^{-1} and 4000 cm^{-1} were performed. Spectra were normalized at 1107 cm^{-1}, the approximate wavenumber of C-O-C bonds in cellulosic substrates. The most representative spectra were chosen for discussion.

Along with the usual cellulose signals, slight differences in peak shape of FTIR signals near 3300 cm^{-1} (O-H), 1400 cm^{-1} (C-H bending, C-OH stretching), 900 cm^{-1} (group C_1 frequency), and 1650 cm^{-1} (adsorb water) between CNF-t to CNF-fur could be observed (see Fig. S6). The signal change around 1400 cm^{-1} is due to crystallinity differences between the two samples, which is supported by the change in the C_1 group frequency near 900 cm^{-1}.[3] The change in crystallinity between the two samples is also supported by the ssNMR results with corresponding changes in C4 (at 84 ppm) and C6 (at 63 ppm) resonances (see Fig. 3 in manuscript).

The expected amide signal should appear near 1570 cm^{-1}(N-H) and 1640 cm^{-1} (CONH). FTIR is able to see changes in peak shape in this region (1560-1700 cm^{-1}), but they cannot be used as a proof of the amidation reaction between furfurylamine and surface carboxylic acid groups, as the expected signals from EDC-urea (carries both the CONH and N-H) and EDC-acylurea (CONR, HN-Et, N-H) lie in the same region (1560 cm^{-1} to 1700 cm^{-1}). Similarly, the changes observed in the signal near 1640 cm^{-1} cannot be unambiguously used to confirm grafting via a covalent bond since it belongs to absorb water molecules in CNF-t,[3,4] whose amount can vary in all three samples.

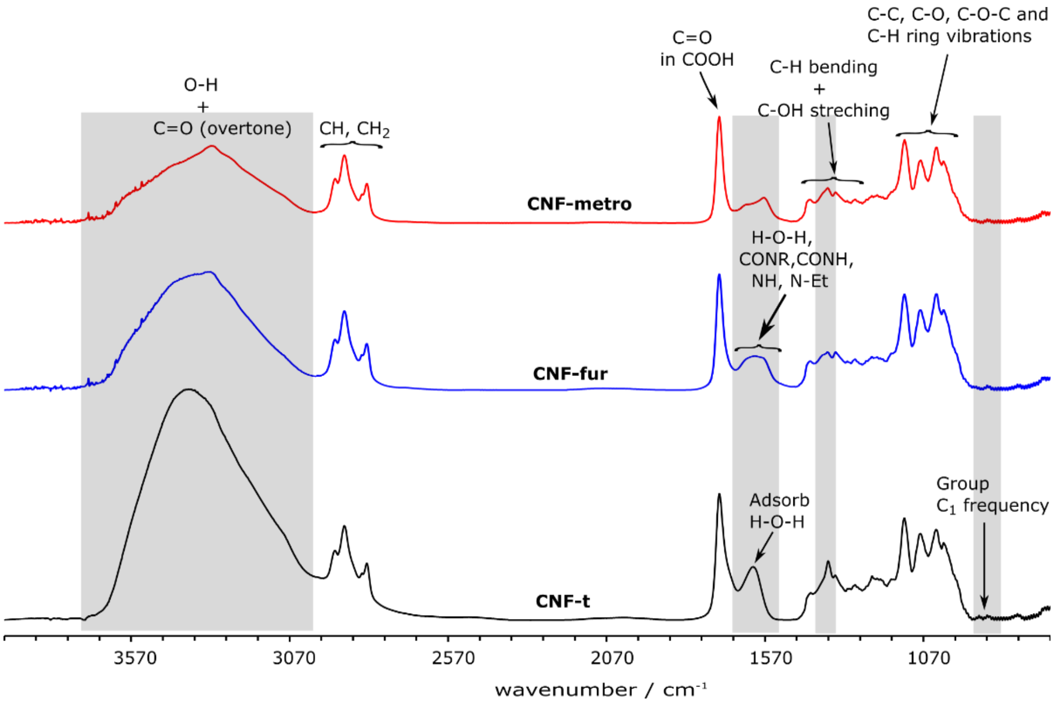

Fig. S6 FTIR spectra for CNF-t (black), CNF-fur after amidation (blue), and CNF-metro after Diels-Alder reaction (red) with the corresponding signal assignment. The shaded regions highlight signals with changes in the three spectra. All spectra were measured through KBr disc preparation.

4.3. ELEMENTAL ANALYSIS

CNF films were prepared by solvent casting from CNF suspensions. The suspensions were poured into Teflon molds and evaporated overnight in an oven at 40°C. Elemental Analysis was performed on a vario Micro Cube® device from Elementar. Carbon, Hydrogen, Nitrogen, Sulfur and Oxygen mass proportions of CNF films were measured. Film pieces of 4 to 7 mg were weighted on a micro-balance. An average of four measurements were obtained for each sample.

Table S1. Results of elemental analysis for mass proportion of carbon, hydrogen, nitrogen, and sulfur of CNF films.

	% C	*sd*	% H	*sd*	% N	*sd*	% S	*sd*	% O	*sd*
**CNF-t**	**35.82**	*0.18*	**5.69**	*0.15*	**< 0.10**	-	**< 0.10**	-	**52.11**	-
**CNF-fur**	**39.23**	*0.68*	**6.12**	*0.12*	**0.47**	*0.01*	**< 0.10**	-	**51.66**	-
**CNF-metro**	**39.78**	*0.02*	**6.12**	*0.03*	**0.68**	*0.01*	**< 0.10**	-	**50.55**	*0.03*

5. DNP-ENHANCED SOLID-STATE NMR SPECTROSCOPY

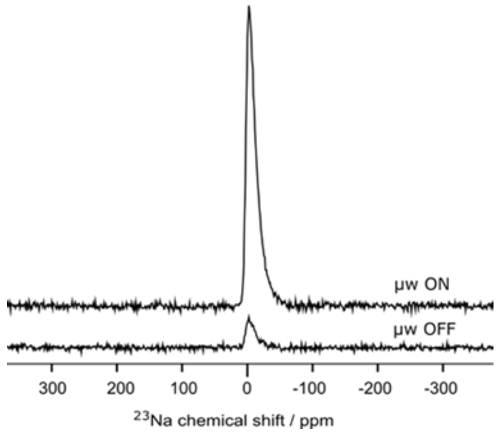

Fig. S7 ^{23}Na CPMAS NMR spectra with and without the application of microwave irradiation suitable for DNP enhancement on CNF-t.

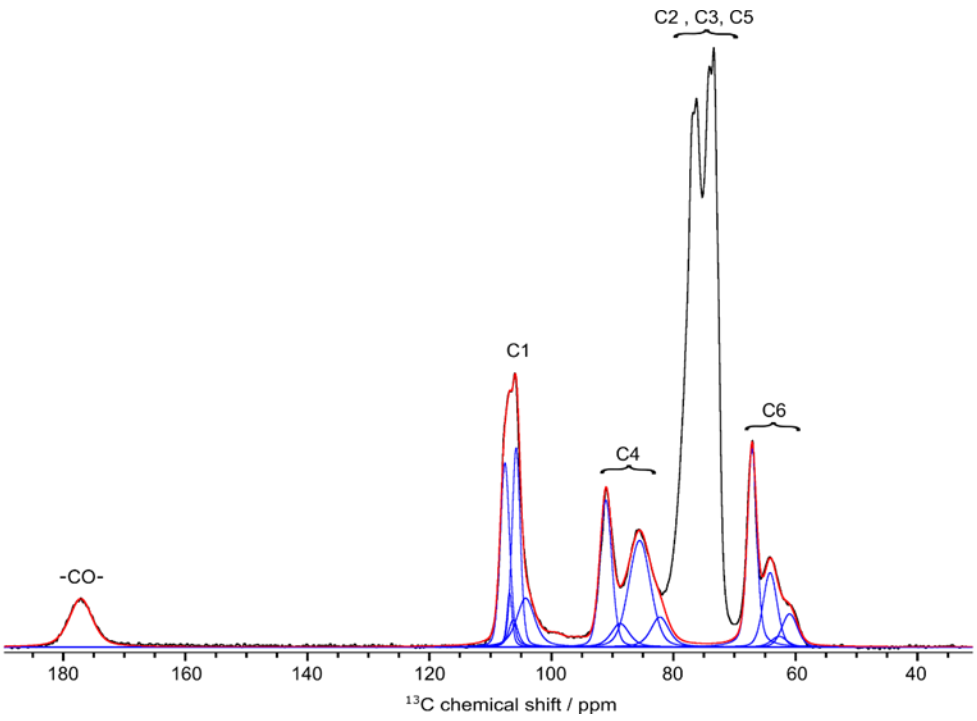

Fig. S8 ^{13}C multi-CP[5] NMR spectra of CNF-t, shown in black, with peak deconvolution of C6, C4, C1, and carbonyl resonance shown in blue. Resultant sum of deconvolution is shown in red. Error (in % of C4 integral) for separate peak was estimated to 0.2 based on average noise, and to 1 in case of deconvolution.

Table S2. Amount in % of different carbons obtained from integration of MultiCP NMR spectra of CNF-t.

Carbon species	Amount (%)	Error (%)
C4	100	1
C1	101	1
C6	74	1
COO^-	24	0.2

REFERENCES

1 D. da Silva Perez, S. Montanari and M. R. Vignon, *Biomacromolecules*, 2003, **4**, 1417–1425.

2 F. Hoeng, A. Denneulin, C. Neuman and J. Bras, *J. Nanoparticle Res.*, 2015, **17**, 17:244.

3 F. Carrillo, X. Colom and J. J. Su, 2004, **40**, 2229–2234.

4 P. Fei, L. Liao and B. Cheng, 2017, 6194–6201.

5 R. L. Johnson and K. Schmidt-Rohr, *J. Magn. Reson.*, 2014, **239**, 44–49.
